# LONGEVITY POLYGENIC RISK IN MULTIDOMAIN AGING AND LONG-TERM SURVIVAL AMONG OLDER WHITE AND BLACK ADULTS

**DOI:** 10.1093/geroni/igae098.2810

**Published:** 2024-12-31

**Authors:** Siting Chen, Corey L Nagel, Jodi Lapidus, Ana Quiñones

**Affiliations:** OHSU-PSU School of Public Health, Portland, Oregon, United States; University of Arkansas for Medical Sciences, Little Rock, Arkansas, United States; OHSU-PSU School of Public Health, Portland, Oregon, United States; Oregon Health & Science University, Portland, Oregon, United States

## Abstract

This study aims to identify genetic characteristics of multi-dimensional aging and longevity by examining the association of longevity polygenic risk scores with multi-domain aging trajectories and long-term survival in older Non-Hispanic White and Non-Hispanic Black adults. We studied 7,287 older adults (≥ 65 years, 14.0% Non-Hispanic Black) from the Health and Retirement Study (HRS, 1998-2016). Group-based trajectory modeling was performed to identify distinct groups of older adults following similar joint trajectories of aging across four domains (multimorbidity burden, functional status, cognitive performance, and depressive symptomatology). We identified four distinct multi-domain trajectory groups: minimal impairment with low multimorbidity (34.1%), minimal impairment with high multimorbidity (37.1%), multidomain impairment with intermediate multimorbidity (16.7%), and multidomain impairment with high multimorbidity (12.1%). Longevity polygenic risk scores (categorized as low, medium, medium-high, and high) were compared between long-term survival groups (long-lived group and controls) across the identified trajectory groups in Non-Hispanic White and Black adults. The proportion of long-lived group was significantly higher in minimal impairment with low multimorbidity group in both Non-Hispanic White (23.3%,p< 0.01) and Non-Hispanic Black adults (19.6%,p< 0.01). The long-lived group had greater proportions of Non-Hispanic White participants with high longevity polygenic risk scores in all trajectory groups. The long-lived group had lower proportions of Non-Hispanic Black participants with low longevity polygenic risk scores in all trajectory groups except for the multi-domain impairment with high multimorbidity group. Our study highlights the need to examine the constellation of genetic and socioeconomic factors on aging-related health domains and longevity among diverse groups of older adults.

